# Strategies for monitoring and managing mass populations of toxic cyanobacteria in recreational waters: a multi-interdisciplinary approach

**DOI:** 10.1186/1476-069X-8-S1-S11

**Published:** 2009-12-21

**Authors:** Andrew N Tyler, Peter D Hunter, Laurence Carvalho, Geoffrey A Codd, J Alex Elliott, Claire A Ferguson, Nick D Hanley, David W Hopkins, Stephen C Maberly, Kathryn J Mearns, E Marion Scott

**Affiliations:** 1 School of Biological and Environmental Science, University of Stirling, FK9 4LA, UK; 2 Centre for Ecology and Hydrology, Bush Estate, Penicuik, Midlothian, EH26 0QB, UK; 3 Division of Molecular Microbiology, School of Life Sciences, University of Dundee DD1 5EH, UK; 4 Centre for Ecology and Hydrology, Lancaster Environment Centre, Library Avenue, Bailrigg, Lancaster, LA1 4AP, UK; 5 Department of Statistics, University of Glasgow, Glasgow, G12 8QW, UK; 6 Department of Economics, University of Stirling, Stirling, FK9 4LA, UK; 7 Scottish Crop Research Institute, Invergowrie, Dundee, DD2 5DA, UK; 8 School of Psychology, College of Life Sciences and Medicine, University of Aberdeen, Aberdeen, AB24 2UB, UK

## Abstract

Mass populations of toxin-producing cyanobacteria commonly develop in fresh-, brackish- and marine waters and effective strategies for monitoring and managing cyanobacterial health risks are required to safeguard animal and human health. A multi-interdisciplinary study, including two UK freshwaters with a history of toxic cyanobacterial blooms, was undertaken to explore different approaches for the identification, monitoring and management of potentially-toxic cyanobacteria and their associated risks. The results demonstrate that (i) cyanobacterial bloom occurrence can be predicted at a local- and national-scale using process-based and statistical models; (ii) cyanobacterial concentration and distribution in waterbodies can be monitored using remote sensing, but minimum detection limits need to be evaluated; (iii) cyanotoxins may be transferred to spray-irrigated root crops; and (iv) attitudes and perceptions towards risks influence the public's preferences and willingness-to-pay for cyanobacterial health risk reductions in recreational waters.

## Background

Cyanobacteria pose short- and long-term risks to human health when growing as mass populations (blooms, scums, biofilms) because they can produce several potent toxins [1,2]. These so-called cyanotoxins include neurotoxic, hepatotoxic, genotoxic, inflammatory and cytotoxic agents. Microcystins are among the most potent and commonly encountered [1]. These toxins constitute one of the highest risk categories of waterborne toxic biological substances, as shown by: (i) the annual occurrence of toxic cyanobacterial populations in water bodies used for drinking, recreation, stock-watering, fisheries and crop irrigation; (ii) episodes of illness and mortality attributed solely or partly to cyanotoxins; and (iii) the identification by national and international bodies of a need for improved risk management to protect water resources, water-based economies and human health [3-6]. Mass populations of toxic cyanobacteria are a global phenomenon and the recent recognition that incidences of blooms may increase significantly under future climate change serves to reinforce further the seriousness of the potential risks to human health [7]. Yet, strategies for monitoring and managing cyanobacteria blooms tend to be reactionary and we currently lack a proactive early warning capability.

Here we propose that a multi-interdisciplinary approach incorporating the natural and social sciences is essential if we are to protect adequately animal and human health from the risks posed by mass populations of toxic cyanobacteria. This paper summarises the results of a multi-interdisciplinary study, using a tiered risk assessment strategy to: (i) develop statistical and process-based models to predict the occurrence of toxin-producing cyanobacteria in waterbodies; (ii) evaluate techniques for the detection and identification of cyanobacterial cells and toxins in field samples; (iii) develop remote sensing as a tool for bloom early-warning monitoring; (iv) evaluate the potential for cyanotoxin transfer to spray-irrigated root crops; and (v) undertake a socio-economic study of public awareness and perceptions of the health risks associated with mass populations of toxic cyanobacteria and their preferences and WTP for risk reductions.

## Methods

### National and local-scale models for cyanobacterial risk assessment

Knowledge of which lakes are susceptible to the development of large populations of potentially-toxic cyanobacteria can be used to inform national-scale assessments of risks to public health. Statistical approaches have been previously used to model the response of phytoplankton communities to nutrient enrichment and reduction [8,9]. However, in this study, a statistical model was developed to specifically predict the occurrence of toxic cyanobacteria genera in UK lakes. The dataset was comprised of 262 phytoplankton samples drawn from observations at 134 lakes. General additive models for predicting the presence/absence of toxic cyanobacteria or their abundance (% relative abundance or biovolume) were developed from the knowledge of widely-available lake parameters (altitude, mean depth, alkalinity, colour, retention time and TP concentration) [10].

The process-based PROTECH model was used to examine the local-scale impacts of climate and land-use change on the occurrence of potential toxin-producing cyanobacteria in two water bodies: Esthwaite Water and Loch Leven (UK). Model scenarios considered the effects of different temperature, nutrient-loading and flushing regimes on the seasonal abundance of toxic cyanobacterial genera. Further details of the PROTECH modelling can be found in [11,12].

### Early-warning monitoring using airborne remote sensing

The retrieval of Chl *a *concentrations from remotely sensed data has been widely used to monitor the development of phytoplankton blooms in inland, coastal and ocean waters. However, this approach does not allow the biomass of cyanobacteria to be determined independently from that of the total phytoplankton standing crop. It has been recently shown that algorithms for the retrieval of the cyanobacterial biomarker pigment C-PC can be used to determine the abundance of cyanobacteria in lakes [13-17]. A comprehensive review of progress in this field can be found in [18].

In this study, CASI and AISA Eagle and Hawk data were collected over Esthwaite Water on 26 April 2007 and Loch Leven on 13 April and 22 August 2007. CASI was operated in Spatial Mode using the band configuration detailed in [16]. AISA Eagle and Hawk are tandem hyperspectral instruments with 244 and 240 contiguous bands distributed across the 400-970 nm and 1000-2400 nm ranges respectively. The airborne data were atmospherically corrected to *R*_rs _using the FLAASH model. The Hawk data were used for aerosol and water vapour retrieval. Algorithms for the retrieval of Chl *a *and C-PC were then derived semi-empirically by regressing near-infrared-to-red band-ratios against measured pigment concentrations and comparisons were made to measured cell densities and toxin concentrations.

### Microcystin detection

Cyanobacterial cell counts and microcystin analyses were used to evaluate the potential risks to human health in Esthwaite Water and Loch Leven. Cells counts were undertaken according to standard procedures using an inverted-microscope. MC concentrations in a cyanobacterial cell-containing particulate fraction and as dissolved toxins in the water were determined by PDA-HPLC and immunoassay [19].

### Toxin transfer to spray-irrigated crops

The potential for toxin transfer to spray irrigated crops [20] was evaluated through a greenhouse experiment. Replicate potato plants were grown with spray-irrigation using water spiked with purified MC-LR at the following concentrations: 0, 1.26, 12.6, 126 μg L^-1^. Plant leaves were harvested at intervals and the entire plant was then destructively harvested at maturity. Samples of roots, tubers, shoots and leaves and soil were freeze-dried and subsequently toxin concentrations were measured in the plant tissues and soil using ELISA with verification by PDA-HPLC.

### Risk perception and CV of cyanobacterial health risks

Non-use values are important components of the economic benefits of water quality improvements [21,22]. Therefore, a CV survey including statements about perceptions of risk of local residents and anglers in four small towns bordering Loch Leven was undertaken to determine their WTP for reductions in the risks to human health posed by blooms of toxin-producing cyanobacteria.

## Results and discussion

### National and local-scale models for risk assessment

**Figure 1:**
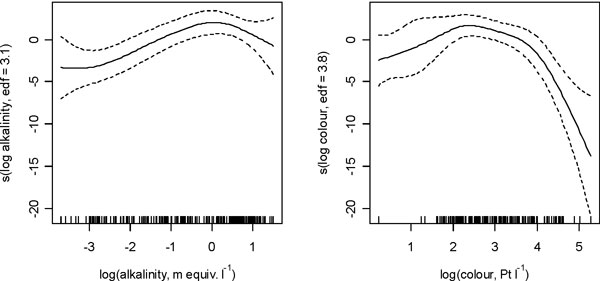
**GAM for predicting the biovolume of toxic cyanobacteria in UK lakes**. The response of log cyanobacterial biovolume to log alkalinity and log colour respectively with dashed lines to indicate ±2 standard errors, edf indicates the estimated degrees of freedom used to fit each variable.

The best model for national-scale risk assessment was developed for log total cyanobacterial biovolume (R^2^_adj _22.3%); both log retention time and log TP concentration showed positive linear relationships with the response, which were borderline significant, with coefficients (standard errors) of 0.590 (0.313) and 0.865 (0.455) respectively. Significant humped relationships were apparent with log colour and log alkalinity (see Figure 1). The models show some predictive ability, but further modelling at an individual lake level would seem necessary. Nevertheless, the results suggest that such models could be applied to targeting lake monitoring and management more efficiently at those lakes at highest risk of breaching WHO guidance levels [see 3].

**Figure 2:**
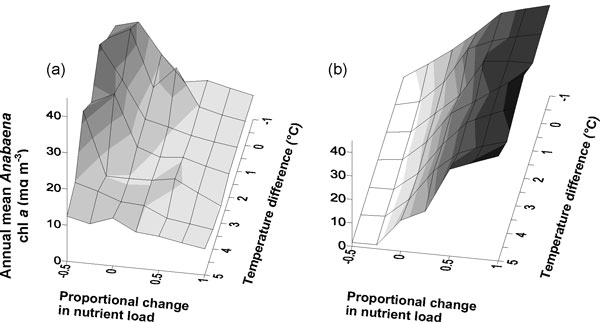
**Mean annual *Anabaena *abundance in Loch Leven**. The response of *Anabaena *(chl *a *mg m^-3^) to changes in nutrient load and temperature in Loch Leven: (a) nitrate and SRP (b) SRP. (Reproduced from [12]).

**Figure 3:**
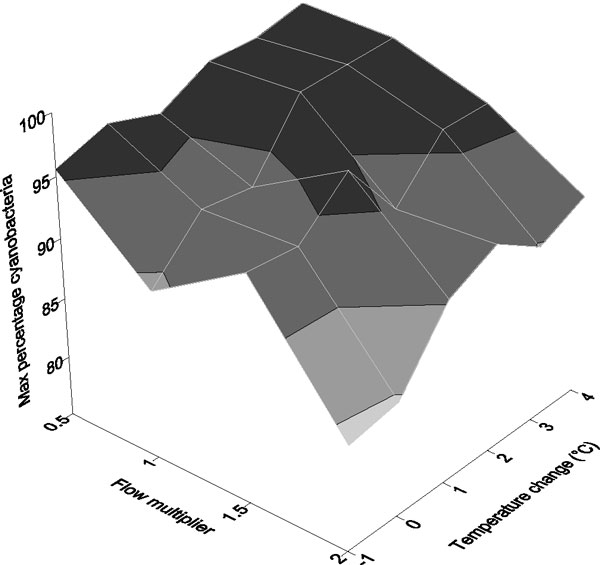
**Summer maximum cyanobacterial abundance in Esthwaite Water**. The response of summer maximum percentage cyanobacterial abundance to changing water temperature (°C) and flushing rate in Esthwaite Water (adapted from [11]).

PROTECH modelling demonstrated that the cyanobacteria in Loch Leven showed great sensitivity to changes in nutrient supply, particularly that of nitrogen which, when reduced, increased the likelihood of blooms (Figure 2). It is likely that this is, in part, because some cyanobacterial species can fix atmospheric nitrogen and thus continue to grow in conditions that would be unfavourable to other species. Water temperature increases had little effect and the lake seemed to be heavily controlled by nutrient resource supply [12]. In contrast, the modelling of Esthwaite Water showed that cyanobacterial bloom formation was enhanced by increasing temperature and decreasing flow (flushing) (Figure 3). However, the specifics of this response were again influenced by nutrient supply and nitrogen availability [11].

### Remote sensing and microcystin analysis

**Figure 4:**
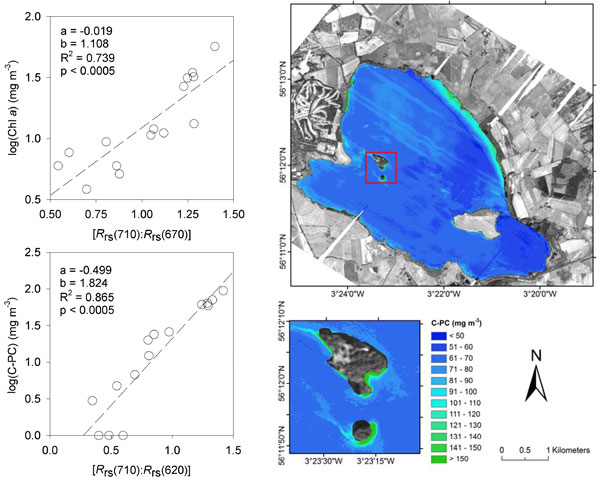
**Scatter plots and concentrations of C-PC in Loch Leven**. Figures showing (a) the relationship between *R*_rs_(710):*R*_rs_(670) and the log concentration of Chl *a*; (b) the relationship between *R*_rs_(710):*R*_rs_(620) and the log concentration of C-PC; and (c) the concentration of C-PC in Loch Leven on 22 August 2007 as retrieved from airborne CASI data using the semi-empirical algorithm (the inset figure shows the presence of a thick cyanobacterial scum on the windward shoreline of Castle Island).

The remote sensing based algorithms demonstrated very strong relationships with the measured pigment concentrations (Chl *a*: R^2 ^= 0.74; C-PC: R^2 ^= 0.87). Figure 4 shows relationships between the near-infrared-to-red band-ratios and the measured pigment concentrations alongside a CASI image of C-PC concentrations in Loch Leven on 22 August 2007. The algorithms were robust when validated against independent datasets. However, for early-warning purposes, further work is required to determine the minimum detection limits of Chl *a *and C-PC and their dependency on the optical properties of inland waters as it is known that concentrations of other optically-active substances (e.g. mineral particles, organic detritus and coloured dissolved organic matter) can affect the accuracy of pigment retrieval.

Microcystins were detected in water samples on all occasions during the airborne sorties, with total MC concentrations (particulate plus soluble) ranging from <1 to >30 μg L^-1^. High correlations between total and particulate MC concentrations versus C-PC were obtained (r = 0.952 and 0.945 respectively), whereas poor agreement occurred between soluble MC concentrations and C-PC (r = 0.459). The results demonstrate that remote sensing has potential as a tool for monitoring blooms of cyanobacteria in inland water bodies, including those containing particulate MCs at concentrations below and exceeding health guidelines [3] and for aiding risk assessment activities [17].

### Microcystin transfer to spray-irrigated crops

Exposure trials have been performed on a range of growing food crop plants and sterile potato shoots using aqueous solutions of MC, including via aerial spraying and root sprinkling [23-25], with multiple adverse outcomes. These include leaf necrosis [23,24] and inhibition of seedling growth [24] and whole-leaf photosynthesis [23]. MCs have been detected in exposed plant tissues in previous studies by ELISA [24,25]. In agreement, we also measured low concentrations of MC (max. 1 ng MC g dry wt^-1 ^plant tissue) by ELISA in potato leaves, roots and tubers in the present trials, but only when potato plants were sprayed with the highest MC concentration (126 μg l ^-1^). Notably, no confirmatory identification of MC in the exposed potato tissues was achieved using PDA-HPLC. Since ELISAs do not distinguish between authentic MC and enzymically-converted MC-detoxication products [26], the question of whether sprayed edible crop plants, including potatoes, may contain non-metabolised, toxicologically-available MC requires further research.

### Risk perception and CV of cyanobacterial health risks

Three hundred and seventy responses to the risk perception and CV questionnaire were received. Fifty one percent were from female respondents. The mean age of respondents was 52 years and the mean combined household income was £50 974 per annum. Thirty four percent of the respondents were unemployed or retried and just over 70% of the sample had received some form of higher education. The results showed that the majority of respondents (66%) believed that there are risks associated with cyanobacterial blooms and 43% did not feel comfortable about these risks. Interestingly, 71% said they were not aware of any specific adverse health effects arising from contact with toxic cyanobacteria, although 44% and 38%, respectively, agreed that there was a low or moderate risk to their own health. The results suggest that the public interviewed has some knowledge about health risks from cyanobacteria but there are inconsistencies in how these risks are evaluated.

**Figure 5:**
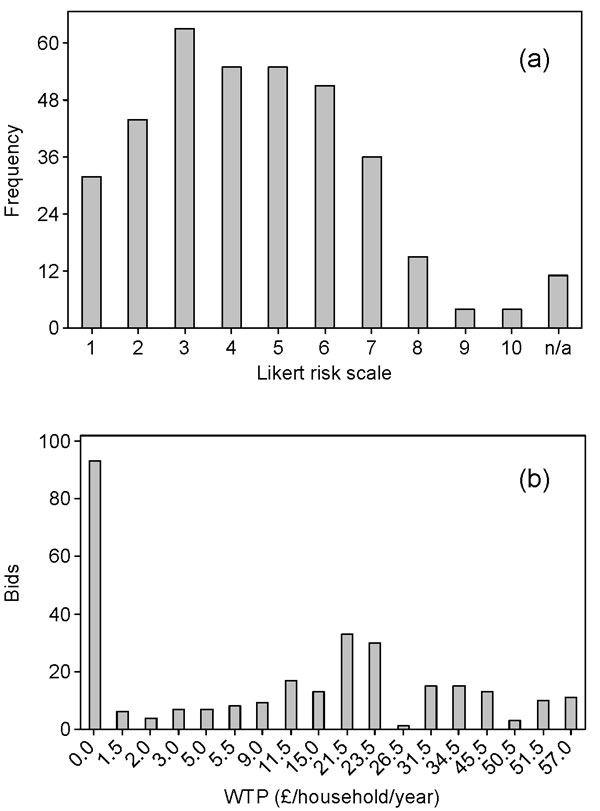
**Risk perception and WTP for health-risk reductions**. Histograms showing (a) the perceived risk from blooms of toxic cyanobacteria in Loch Leven (1 = no risk; 10 = very high risk) and (b) the distribution of WTP bids in relation to a reduction in the number of days on which toxic cyanobacteria present risks to human health in Loch Leven from 90 (status quo) to either 45 or zero days risk.

The majority of respondents rated the health risk posed by toxic cyanobacteria at Loch Leven as low to moderate. However, more than 50% of the sample were willing to pay additional local taxes for measures to reduce these risks by reducing nutrient inputs to Loch Leven (mean = £16.60/household/year; standard error = £1.00/household/year), with higher values being associated with larger risk reductions in terms of the number of days per year when cyanobacteria present risks to humans (Figure 5). Important determinants of variations in WTP were extent of use of the loch, employment, household income and some risk attitude measures.

## Conclusion

Mass populations of toxic cyanobacteria present significant environmental and human health hazards. In this study we demonstrate that it is possible to model water bodies at risk of toxic blooms at a national scale: an approach that could be used to develop a proactive monitoring strategy. PROTECH modelling of two study lakes demonstrated the importance of nutrients to cyanobacteria abundance, but the responses to climate change appear to be more complex. We have shown that remote sensing has the potential to provide timely data for risk assessment activities, but there is a need for further work on the optimisation of algorithms and the assessment of their minimum detection limits. The use of contaminated water for crop irrigation has been shown to be a potential route for toxin transfer to crop plants. However, our results for potatoes remain inconclusive and further work is needed to determine whether edible plants may contain toxicologically-available MCs. The results from the risk perception and CV study indicated that the general public has a limited knowledge of the health risks associated with cyanobacteria, but their perception of the risks determines their preferences and WTP for risk management activities.

## List of abbreviations used

CASI: compact airborne spectrographic imager; Chl *a*: chlorophyll a; C-PC: cyanobacterial phycocyanin; CV: contingent valuation; DGGE: denaturing gradient gel electrophoresis; EDF: estimated degrees of freedom; ELISA: enzyme-linked immunosorbant assay; GAM: generalised additive model; HPLC: high performance liquid chromatography; MC: microcystin; PDA: photodiode array; PROTECH: phytoplankton responses to environmental change; RMSE: root mean square error; *R*_rs_: remote-sensing-reflectance; SRP: soluble reactive phosphorus; TP: total phosphorus; WHO: World Health Organisation; WTP: willingness-to-pay.

## Note

The peer review of this article can be found in Additional file 1.

## Competing interests

The authors declare that they have no competing interests.

## Authors' contributions

ANT conceived and designed the study and was involved in the acquisition and analysis of the remote sensing data and helped design the CV survey. PDH was involved in the design of the study, waterbody sampling and analysis, acquisition and analysis of the remote sensing and the design, administration and analysis of the CV survey. LC jointly led the national scale risk monitoring, cyanobacteria cell counts and contributed to the design of the CV study. GAC was involved in the design of the study, conducted toxin analyses, and assisted with the design of the CV survey. JAE led and undertook the PROTECH modeling. CAF undertook the national scale risk modeling. NDH led on the CV survey. DWH jointly designed and led the spray irrigation experiments. SCM led on the PROTECH modeling and assisted with waterbody sampling and cyanobacteria cell counts. KJM led the risk perception study. EMS jointly led on the national scale modeling. All authors contributed to and approved the drafting of the final manuscript.

## Supplementary Material

Additional file 1 Peer review Click here for file
