# Supplementary material for: Strategies for monitoring and managing mass populations of toxic cyanobacteria in recreational waters: a multi-interdisciplinary approach
Source: Environ Health. 2009 Dec 21;8(Suppl 1):S11. doi: 10.1186/1476-069X-8-S1-S11 (PMC2796489; doi:10.1186/1476-069X-8-S1-S11)
Supplement: Additional file 1 — Peer review [file 1476-069X-8-S1-S11-S1.doc]

**SCHOOL OF BIOLOGICAL AND ENVIRONMENTAL SCIENCES**

**Professor David Goulson**

**Head of School**

University of Stirling

Stirling

FK9 4LA Scotland

Telephone: +44 (0) 1786 467840

Facsimile: +44 (0) 1786 467843

Professor Michael Moore

Science Co-ordinator - Joint Environment & Human Health Programme

Plymouth Marine Laboratory

Prospect Place, The Hoe

Plymouth

PL1 3DH, UK

30 November 2026

**Re: Paper submitted for inclusion in the Proceedings of 2nd Annual Conference of the Environment & Human Health Programme to be published in "Environmental Health"**

Dear Professor Moore,

We appreciate the constructive comments received from two anonymous reviewers in regards to the above manuscript. We have now carefully considered all the issues raised and have made appropriate revisions to the manuscript where necessary. We are grateful for the relaxation of the original word limit to enable us to address the points raised by the reviewers. For clarity, we include the reviewers’ original comments (*italicised text*) with our subsequent responses and revisions detailed beneath (indented text).

Reviewer#1

**General comments:** *This is a potentially useful multi-disciplinary paper tackling cyanobacteria issues, aiming to predict and monitor lakes. The Methods are mostly acceptable, but the Results are lacking most of the details needed to justify and replicate this research. In particular, the results lack: model parameters and relationships; remote sensing algorithm coefficients and plots. Risk perception is lacking a graph. The Methods should refer to previous satellite or airborne remote sensing methods.*

We appreciate that the reviewer recognizes the value of our study to the field. The omission of this material from the original manuscript was largely due to the need to adhere to the prescribed limits of 1500 words, two tables (or figures), and 20 references. The research described in the manuscript is obviously of a very diverse nature and thus it is difficult to provide substantive detail on all components of the research. Where possible we refer the reader to consult other published papers from this research. However, we agree that this additional information is needed to justify and replicate the research outlined in the manuscript.

The methods section of the manuscript has therefore been revised to include a brief review of previous studies using remote sensing for monitoring cyanobacterial blooms (including our own). We also now include (where appropriate) additional figures detailing the parameters and relationships for the local and national scale risk assessment models, scatter plots detailing the remote sensing algorithms and coefficients, and a graph supporting the risk perception work.

*I am unused to seeing a List of abbreviations at the end, but I will leave to the Editor the decision whether to return to the normal definition of abbreviations on their first usage. (At least it should indicate at the start that there is a list of abbrevs.).*

We chose to list the abbreviations at the end of the manuscript to reduce the length of the main body text. This is in line with the instructions for authors provided by *Environmental Health*.

**Major compulsory revisions:**

Methods

P5 *Early-warning monitoring using remote sensing: here or in the Background there should be a brief literature review of other satellite/airborne RS methods for detecting cyanobacteria blooms. I know of a couple by Kahru but there must be others. Also lacking data description, e.g. bands, quantity of data.*

This lack of this information was, as previously discussed, a reflection of the original word limit. In the revised manuscript we provide a brief review of the literature and have added information on the type and quantity of data used as requested. The references cited in this section contain more detailed descriptions of the methods than it is possible to provide in this brief communication. To maintain brevity, we also refer the reader to the recent review article of Kutser (2009) for further information on recent work in this field.

The revised text reads: “The retrieval of Chl *a* concentrations from remotely sensed data has been widely used to monitor the development of phytoplankton blooms in inland, coastal and ocean waters. However, this approach does not allow the biomass of cyanobacteria to be determined independently from the total phytoplankton biomass. It has been recently shown that algorithms for the retrieval of the cyanobacterial biomarker pigment C-PC can be used to determine the abundance of cyanobacteria in lakes [12-16]. A comprehensive review of progress in this field can be found in [17].

In this study, CASI and AISA Eagle and Hawk data were collected over Esthwaite Water on 26 April 2007 and Loch Leven on 13 April and 22 August 2007. CASI was operated in Spatial Mode using the band configuration detailed in [15]. AISA Eagle and Hawk are tandem hyperspectral instruments with 244 and 240 contiguous bands distributed across the 400-970 nm and 1000-2400 nm ranges respectively.”

Results

P6 *National and local scale models: lacking coefficients of the ‘best model’, otherwise readers cannot appreciate or replicate findings. Lacking graph of model fit to data.*

The model coefficients (and standard errors) for the linear covariates are now presented in the main text of the revised manuscript. Those covariates that are smooth terms do not have coefficients; instead, we include a plot of their relationship with the response variable (Fig. 1 in the revised manuscript).

The revised text reads: “The best model for national-scale risk assessment was developed for log total cyanobacterial biovolume (R^2^_adj_ 22.3%); both log retention time and log TP concentration showed positive linear relationships with the response, which were borderline significant, with coefficients (standard errors) of 0.590 (0.313) and 0.865 (0.455) respectively.  Significant humped relationships were apparent with log colour and log alkalinity (see Fig. 1).”

…para 2: *Need data evidence for statement: “PROTECH modelling... demonstrated that…”. Probably needs graph to justify statement “Water temperature increases had little effect…nutrient supply”. Also need data evidence for statement “However, the specifics… nitrogen availability”*

We have included an additional graph showing the effect of nutrient supply on cyanobacteria in Loch Leven that supports our statements in the original text. In the revised manuscript we also now refer the reader to consult the papers by Elliott and May (2008) and Elliott (2009) which present a more detailed discussion of the application of PROTECH to the modeling of cyanobacteria in these lakes.

P7 *Remote sensing: Lacking everything on the RS algorithms: coefficients, scatterplot.*

The semi-empirical algorithms developed and used for the retrieval of Chl *a* and C-PC (including model coefficients) are now shown in Fig. 4 of the revised manuscript.

P8 *Risk perception: Would benefit from a graph of results.*

A graph showing the perceived risk from toxic cyanobacteria and the distribution of WTP bids for cyanobacterial health-risk reductions in Loch Leven is now included in the revised manuscript (Fig. 5).

**Minor essential revisions:**

P2 *Abstract: “spray-irrigated to crops” remove “to”.*

P3 para 1: *“Microcystins,” remove comma.*

P4 para 3: *“considered the effect OF different…”.*

P5 para 2: *“CELL counts…”.*

P6 para 3: *“Fig 2” should be “Fig. 1”.*

Figure 1: *Legend should state year of data.*

These minor revisions have been made to the manuscript as suggested with the exception of the addition of the year to the legend of Fig.1. Fig. 1 (Fig. 3 in the revised manuscript) is derived from the PROTECH modelling and therefore there is no “year” for the data presented.

Reviewer#2

**General comments:** *Harmful algal blooms (HABs) are a worldwide problem of nutrient runoff and contamination of water bodies. The paper addresses the problem in a classical way – the multidisciplinary approach used is not a novel approach.*

We appreciate that the reviewer recognizes that our manuscript deals with a problem of global significance. The multidisciplinary approach we adopt is not entirely new (although we do not believe it can yet be thought of as “classical”). However, the various components that we bring together to tackle this problem, GAM modeling, PROTECH modeling, remote sensing, and CV and risk perception studies, include a great deal of novel research.

**Minor Essential Revisions:**

*In the 2^nd^ paragraph below Methods, beginning with “The process-based PROTECH model….” The 4^th^ line down from there, did the authors mean to write, the effects of different temperature…., etc?*

We have revised this paragraph to read “Model scenarios considered the effects of different temperature, nutrient-loading and flushing regimes on the seasonal abundance of toxic cyanobacterial genera”.

P.4., *6^th^ line down under Methods, requires a brief statement regarding the nature of the methods from references 9 & 10. At least state what the methods employed in these references are.*

The papers by Jeppesen et al. (2005) and Ferguson et al (2008) use a similar statistical approach to that adopted in our study. However, in a departure from these previous studies, we specifically modeled the response of toxic cyanobacteria genera.

We have revised the text to clarify the difference in our approach. The text now reads: “Statistical approaches have been previously used to model the response of phytoplankton communities to nutrient enrichment and reduction [8,9]. However, in this study, a statistical model was developed to specifically predict the occurrence of potentially-toxic cyanobacteria in UK lakes.”

**Major compulsory revisions:**

*Why wasn’t turbidity included as part of the lake parameters? Turbidity is a controlling factor for algal blooms*.

We assume that the reviewer is referring to the parameters used in the national scale risk modelling. The objective of this work was to use knowledge of widely available lake parameters to develop a model for predicting the presence and abundance of toxic cyanobacteria. The modelling used pre-existing monitoring data provided by the UK environment agencies. This dataset unfortunately does not include measurements of turbidity. Moreover, as turbidity is often highly dependent on phytoplankton abundance (the exception being some very shallow lakes with high sediment resuspension), it is likely to be of little value for predictive modelling of lakes at risk of developing blooms of toxic cyanobacteria.

*The authors measured only total P concentration, however the limiting factor in HABs, especially microcystis blooms, is bioavailable reactive P (BRP) or soluble reactive P (SRP). This paper would have been strengthened by this differentiation in P types.*

The relationship with SRP is complicated by the fact that in some lakes it can limit total phytoplankton biomass and for this reason it can be undetectable over range of phytoplankton standing crops. However, it is often in excess in many UK lakes and under these conditions it does not necessary demonstrate a clear relationship with phytoplankton biomass. For these reasons TP is commonly used as the explanatory nutrient variable in phytoplankton models as it has the stronger relationship with algal biomass (e.g. OECD TP-Chl models and more recently the European models for WFD). We therefore considered the use of TP to be a more appropriate variable for use in national-scale risk modelling.

*Airborne data are being used in the USA for the same purposes of early-warning monitoring as described herein. In several studies, phycocyanin pigments correlate with water turbidity, which to the best of this reviewer’s knowledge is not included in the FLAASH algorithm. The role of turbidity in aerial remote sensing should have been discussed in this manuscript.*

The reviewer appears to misunderstand the purpose of the FLAASH model. FLAASH is an atmospheric correction model based upon the widely used MODTRAN4 code and provides a means of computing surface reflectance from at-sensor-radiances measured by airborne or satellite platforms. FLAASH cannot be used to directly retrieve information on the composition of the water column (e.g. pigments, mineral particles and colored dissolved substances). In our study, the retrieval of Chl *a* and C-PC concentrations was achieved through the use of semi-empirical algorithms (implemented independently of FLAASH) once the data had been atmospherically corrected. These algorithms are now detailed in Fig. 4 of the revised manuscript.

The request for a wider discussion of the role of turbidity in airborne remote sensing is also a little confusing. There are several (now rather dated) studies in which remotely sensed data have been used to estimate water turbidity (typically as the Secchi disc depth). Water turbidity is often well correlated with phytoplankton biomass (and therefore Chl *a* and C-PC). However, this is a rather simplistic approach to algal bloom monitoring. The direct retrieval of Chl *a* and C-PC from remotely sensed data provides a means of estimating the biomass of phytoplankton and cyanobacterial in the water column. This approach is far superior to the retrieval of water turbidity as a proxy for phytoplankton abundance.

The accuracy of pigment retrieval is however known to be dependent to some extent on the concentrations of other optically-active constituents in the water column (e.g. mineral particles, organic detritus and colored dissolved organic matter). We have expanded our discussion in the revised manuscript to acknowledge these effects. The revised text reads: “The algorithms were found to be robust when further validated against independent datasets. However, for early-warning purposes, further work is required to determine the minimum detection limits of Chl *a* and C-PC and their dependency on the optical properties of inland waters as it is known that concentrations of other optically-active substances (e.g. mineral particles, organic detritus and coloured dissolved organic matter) can affect the accuracy of pigment retrieval.”

*In the risk perception studies at the top of page 8, what are the confidence limits of the responses? What was the breakdown of the respondents in terms of age, sex, educational level or background?*

We have provided the standard error for the estimate of mean WTP and have included a description of the sample demographics in the main text.

The revised text reads: “Three hundred and seventy responses to the risk perception and CV questionnaire were received. Fifty one percent were from female respondents. The mean age of respondents was 52 years and the mean combined household income was £50 974 per annum. Thirty four percent of the respondents were unemployed or retried and just over 70% of the sample had received some form of higher education…. More than 50% of the sample were willing to pay additional local taxes for measures to reduce risks by reducing nutrient inputs to Loch Leven (mean =£16.60/household/year; standard error = £1.00/household/year).”

A figure is also included in the revised manuscript (Fig. 5) showing the perceived risk from cyanobacterial bloom on Loch Leven and the resulting distribution of WTP bids for health-risk reductions as requested by Reviewer#1.

*The authors should be aware of the work of Crush et al., 2008, Effects of Irrigation with Lake Water Containing Microcystins on Microcystin Content and Growth of Ryegrass, Clover, Rape, and Lettuce, Environ. Toxicol. 246-252, which is not cited in this manuscript. This very recent study is actually of greater sophistication and more revealing than the greenhouse study performed in the present manuscript. Various studies have been done on a myriad of vegetable types, it is well known that MC can be transferred from contaminated irrigation water. The potato has been studied in this light. The authors are encouraged to read the studies of McElhiney et al., 2001, Toxicon, 39:1411-1420, who also studied the potato. The authors should cite and discuss their greenhouse findings with potato in light of McElhiney et al., 2001.*

The authors were aware of the studies cited by the reviewer. We did not make reference to these papers in the original manuscript simply because of word constraints. In the revised manuscript we now discuss the results and implications of our exposure trials in the context of these previous experiments. However, it should be noted that, in a departure from the study by McElhiney et al. (2001), we tested for the presence of toxins in potato tubers following exposure in a manner representative of that which occurs in conventional horticultural systems (the foregoing study looked at the effect of toxin exposure on shoot growth and development).

The revised text reads: “Exposure trials have been performed on a range of growing food crop plants and sterile potato shoots using aqueous solutions of MC, including via aerial spraying and root sprinkling [23-25], with multiple adverse outcomes. These include leaf necrosis [23,24] and inhibition of seedling growth [24] and whole-leaf photosynthesis [23]. MCs have been detected in exposed plant tissues in previous studies by ELISA [24,25]. In agreement, low concentrations of MC (max. 1 ng MC g dry wt^-1^ plant tissue) were detected by ELISA in potato leaves, roots and tubers in the present trials , though only when potato plants were sprayed with the highest MC concentration (126 µg l ^-1^). Notably, no confirmatory identification of MC in the exposed potato tissues was achieved using PDA-HPLC. Since ELISAs do not distinguish between authentic MC and enzymically-converted MC-detoxication products [26], the question of whether sprayed edible crop plants, including potatoes, may contain non-metabolised, toxicologically-available MC requires further research.”

We feel our subsequent revisions have improved the original manuscript. We thank you for the opportunity to submit a revised version of our manuscript and look forward to hearing from you in due course.

Yours sincerely,

**Dr Andrew N. Tyler (& Colleagues)**

Reader

School of Biological and Environmental Science

University of Stirling

Stirling, FK9 4LA
